# Analytical Validation of Two Point-of-Care Assays for Hematological Analysis in the Miranda Donkey

**DOI:** 10.3390/vetsci11090450

**Published:** 2024-09-22

**Authors:** Céline Costa, Ana Patrícia Sousa, Grasiene Silva, Felisbina Queiroga, Ângela Martins, Daniela Andrade, Ana C. Silvestre-Ferreira

**Affiliations:** 1 Departamento de Ciências Veterinárias, Universidade de Trás-os-Montes e Alto Douro (UTAD), 5000-801 Vila Real, Portugal; celine.costa99@hotmail.com (C.C.);; 2 Centro de Ciência Animal e Veterinária (CECAV), Universidade de Trás-os-Montes e Alto Douro (UTAD), 5000-801 Vila Real, Portugal; 3 Laboratório Associado para a Ciência Animal e Veterinária–AL4AnimalS, 1300-477 Lisboa, Portugal; 4 Centre for the Research and Technology of Agro-Environmental and Biological Sciences (CITAB), 5000-801 Vila Real, Portugal; 5 Departamento de Zootecnia, Universidade de Trás-os-Montes e Alto Douro (UTAD), 5000-801 Vila Real, Portugal; 6 Associação para o Estudo e Proteção do Gado Asinino (AEPGA), Largo da Igreja, nº 48, 5225-011 Atenor, Portugal

**Keywords:** donkey, HemoCue WBC DIFF, HemoCue Hb 201, Asinina de Miranda

## Abstract

**Simple Summary:**

The Miranda’s donkey, native to Miranda do Douro, Portugal, is an endangered breed. Researchers aimed to validate two portable blood analyzers, the HemoCue WBC DIFF and HemoCue Hb 201, for use in this breed. These instruments are important because they can evaluate white blood cells and measure hemoglobin levels, which are crucial for assessing animal health and welfare. The study tested the precision and accuracy of these analyzers using donkey blood samples. Most results for the HemoCue WBC DIFF were accurate, although there were some issues with certain white blood cells (monocytes and eosinophils). The HemoCue Hb 201 showed very consistent results for measuring hemoglobin. Both instruments proved to be reliable and accurate tools for hematological analysis. Compared to a standard laboratory method, they performed very well for most white blood cells and hemoglobin. However, they were less accurate for less-represented white blood cells. Overall, the study concludes that these portable analyzers are suitable for use in the field and can help veterinarians monitor the health of Miranda donkeys more easily. This is valuable because it helps protect an endangered breed by ensuring timely and accurate health assessments.

**Abstract:**

Miranda donkeys are an endangered autochthonous breed of Portugal. The HemoCue WBC DIFF and HemoCue Hb 201 portable analyzers, developed as a simplified alternative method for total and differential WBC count and hemoglobin measurement, respectively, may be relevant tools in veterinary practice. This study aimed to validate these instruments using Miranda donkey blood samples. For the HemoCue WBC DIFF, most parameters showed acceptable intra- and inter-assay precision with coefficients of variation (CV) below 5%, except for monocytes and eosinophils with higher CVs. The HemoCue Hb 201 showed CVs of 1.98% and 4.07%. Linearity correlation coefficients (r) ranged from 0.53 to 0.99 for HemoCue WBC DIFF and 0.99 for HemoCue Hb 201. Significant levels for neutrophils, lymphocytes, monocytes, eosinophils, and Hb measurements varied. Comparisons with ProCyte Dx showed an excellent correlation for WBC (r_s_ = 0.96), neutrophils (r_s_ = 0.91), lymphocytes (r_s_ = 0.94), and eosinophils (r_s_ = 0.90) but a poor correlation for monocytes and basophils. The HemoCue Hb 201 showed a correlation of r_s_ = 0.96 with ProCyte Dx. In conclusion, both analyzers provided reliable results and are suitable for use in Miranda’s donkey breed for WBC counts and Hb measurements.

## 1. Introduction

In Europe, the population of donkeys (*Equus asinus*) is found mainly in Mediterranean countries, such as Portugal, Spain, France, Italy, and Greece. However, over the past few decades, the number of animals has decreased by approximately 78%, raising significant concerns about both welfare and conservation [1,2,3].

In Miranda do Douro, located in northeastern Portugal, one of the last autochthonous donkey breeds officially recognized within the national territory is preserved: the Miranda Donkey, also known as the Asinina de Miranda breed [4]. Over the years, the population of this breed has declined and is now considered to be endangered. To protect and preserve this breed, not only as a genetic heritage but also as a significant cultural symbol, the Association for the Study and Protection of Donkey Cattle (AEPGA) was established on 9 May 2001, in Portugal [5,6]. Efforts to promote the welfare and conservation of the Miranda Donkey, along with the fact that these animals live far from veterinary facilities or laboratories, have prompted the search for diagnostic tools for analysis on-site/in-field/in-farm, allowing faster and more accessible veterinary care.

In health care, any laboratory testing performed outside the conventional laboratory and in close proximity to the patient is referred to as point-of-care testing (POCT) [1,7]. The HemoCue Hb 201(Radiometer Medical ApS) is a portable device designed to measure the hemoglobin concentration (Hb) in whole blood. Hemoglobin levels provide an indication of oxygen-carrying capacities and is one of the most commonly used biomarkers, alongside red blood cell counts, hematocrit, and mean corpuscular volumes, to assess the prevalence of anemia in humans and animals [8]. The HemoCue WBC DIFF (Radiometer Medical ApS) is a portable, image-based hematology analyzer that uses disposable microcuvettes containing staining and lysis reagents. It performs a total white blood cell (WBC) count and a five-part differential, identifying neutrophils, lymphocytes, monocytes, eosinophils, and basophils. White blood cells play a critical role in the immune system, being essential for defending against inflammation and infection [9,10].

Therefore, this study aims to evaluate the precision (both intra- and inter-assay), linearity, and comparison (using ProCyte Dx-IDEXX and the manual differential count technique) of the POCT system’s HemoCue WBC DIFF for total and differential white blood cell counts, and HemoCue Hb 201 for hemoglobin concentration in Asinina de Miranda breed animals, following the guidelines established by the American Society of Veterinary Clinical Pathology (ASVCP) and the International Council for Standardization in Hematology (ICSH) for the evaluation of blood cell analyzers [7,11,12].

## 2. Materials and Methods

### 2.1. Samples

For this study, whole blood samples were collected from 44 non-fasted Asinina de Miranda breed animals via jugular venipuncture using a vacuum collection system with a 21G needle and 4.0 mL EDTA-K3 tubes (BD Vacutainer). Five samples from healthy animals were used for precision and linearity, and the remaining 39 (10 sick and 29 healthy animals) were used for the comparison study. Samples were transported at a refrigeration temperature, and all procedures were initiated within 1–2 h post-collection, ensuring proper homogenization and adherence to the manufacturer’s instructions. The samples were obtained as part of a prophylactic program developed for the breed, with the animals’ health status regularly monitored by veterinarians from the AEPGA association. All animals in the study received routine anthelmintic treatment following a selective protocol [13,14]. No samples were specifically collected for the purposes of this study, and any samples showing signs of hemolysis or lipemia were discarded.

All owners provided informed written consent for the use of data and any remaining blood samples from their animals. The analyses were performed at the Clinical Pathology Laboratory (LPC) of the Veterinary Teaching Hospital of the Universidade de Trás-os-Montes e Alto Douro (Vila Real, Northern Portugal). This study was approved by the Ethics Committee for Animal Welfare (ORBEA) of the Universidade de Trás-os-Montes e Alto Douro (i467-e-CECAV-2022).

### 2.2. HemoCue

The HemoCue Hb 201 measures Hb concentrations using a small sample (10 μL) of capillary or venous blood. This portable system uses a modified version of Vanzetti’s reagents, employing an azidemethemoglobin reaction [15]. Once the blood is drawn into the microcuvette by capillary force, the erythrocytes are hemolyzed, releasing Hb. The iron present in Hb is then converted from the ferrous to the ferric state by sodium nitrate, forming stable azidemethemoglobin, which is detected by the photometer [15,16,17].

The HemoCue WBC DIFF provides total and differential leukocyte counts, including neutrophils, lymphocytes, monocytes, eosinophils, and basophils [9]. The system operates by using a microcuvette with a small volume of capillary or venous blood (10 µL). The blood sample is drawn into the microcuvette by capillary action, where it interacts with a cell lysis agent that hemolyzes the erythrocytes, while methylene blue dye stains the nuclei of the white blood cells. The microcuvette is then inserted into the analyzer, where the white blood cells are counted through image analysis using a microscopic image detector [10,18,19,20,21].

### 2.3. IDEXX Procyte Dx

The ProCyte Dx, previously validated for studying the hematology of Asinina de Miranda donkeys [22], is an automated cell analyzer designed for veterinary use. It combines three technologies: laser flow cytometry, optical fluorescence, and laminar flow impedance. For Hb measurement, the ProCyte Dx uses the SLS-hemoglobin method [22]. Quality control (ProCyte Dx quality control L1 and L2) was performed before all analyses.

### 2.4. Manual Techniques

For the comparison study, a manual leucocyte differential count was performed. From each sample two blood smears were prepared. The smears stained by Diff-Quik were used for manual leucocyte differential count. A general assessment of the blood smear was performed in the 10x objective, and leucocyte differential count was performed by counting 200 cells in the immersion objective and differentiated into 5 populations: neutrophils (NEU), eosinophils (EOS), basophils (BASO), monocytes (MONO), and lymphocytes (LYM); for each sample, the final result was obtained by counting the same smear twice by two different experienced operators (A.C.S-F., and F.Q.) and the average for each cell type was calculated. Whenever a coefficient of variation (CV%) > 25% was observed between the two measures, smears were examined again to rule out any observer’s technical error [22,23].

### 2.5. Method Validation

#### 2.5.1. Intra-Assay Precision

For the study of intra-assay precision, a sample of one animal was used. For each HemoCue, twenty consecutive measurements were performed in a 2 h time interval by the same operator and under the same conditions, with 2 min rest periods between each measurement, to avoid overheating the devices and, consequently, possible errors [11].

#### 2.5.2. Inter-Assay Precision

For inter-assay precision studies, 5 blood samples were analyzed, following the same procedure for both devices. Each sample was analyzed in quadruplicate within 3 days. These samples were placed randomly and always interspersed with a random blood sample from another animal that entered the Clinical Pathology Laboratory at the Veterinary Hospital of UTAD (LPC-HVUTAD). There was a rest period of approximately 2 min between each measurement to avoid overheating the equipment. Intra-assay and inter-assay precision were performed by determining the coefficient of variation [11,24].

#### 2.5.3. Linearity

For linearity studies, 2 blood samples from the same animal were used. Samples were centrifuged at room temperature at 2618 g for 5 min. Then, the plasma from one of the tubes was removed and transferred to a 1 mL aliquot, which was considered level 1. The buffy coat and the erythrocytes from that same tube were separated into another 1 mL aliquot and were considered level 5, the highly concentrated sample. Five levels of dilution (100, 75, 50, 25, and 0%) were then prepared with the autologous plasma and measured in duplicate in both devices with a rest period of approximately 2 min to avoid overheating the equipment [11,25]. Linearity was obtained by calculating the Pearson correlation coefficient (r) and the level of significance (p) to verify the statistical validity and proceed with the simple linear regression [11,26].

#### 2.5.4. Comparison Study

The comparison study of HemoCue WBC DIFF and HemoCue Hb 201 was performed using 29 and 39 blood samples from Asinina de Miranda donkeys, respectively. For 10 samples, there was not enough of the samples to perform a comparison of both instruments. The samples included sick (*n* = 10) and healthy (*n* = 19/*n* = 29) animals randomly selected from a heterogeneous population regarding sex [(19 females, 20 males (18 castrated)], age, (average 9.8 ± 5.7 years) and animal health status [healthy (*n* = 29); sick (*n* = 10; 7 with diverse inflammatory conditions, and 3 with tumors)]. HemoCue WBC DIFF and HemoCue Hb 201 results were compared to ProCyte Dx (IDEXX). A comparison with the manual count method for the WBC differential count was also performed.

### 2.6. Statistical Analysis

Microsoft^®^ Excel (office 2019) was used to perform descriptive statistics (average values, standard deviation, and coefficient of variation (CV%). To calculate the average CV, for each parameter, each blood sample CV was initially determined. JMP^®^ version 17.2.0 (SAS Institute Inc., Cary, NC, USA, 1989–2023) was employed to calculate the Pearson correlation coefficient (r), the significance level (p), trace linear regressions, Spearman rank correlation, Passing–Bablok regressions, and Bland–Altman plots. Spearman’s Rho (r_s_) was considered excellent when r_s_ = 0.93–0.99, good for r_s_ = 0.80–0.92, fair for r_s_ = 0.59–0.79, and poor for r_s_ < 0.59 [17,21]. Results were considered acceptable when fair and above Spearman’s rank correlation coefficient were observed alongside linear regressions in which the 95% confidence interval (CI) included 1 for the slope and 0 for the intercept [27,28,29].

## 3. Results

### 3.1. Precision Results

Intra- and inter-assay precision results are displayed in Table 1.

In the study performed on the HemoCue WBC DIFF, the CV for most of the results, of both intra- and inter-assay precision, were below the allowed 5%, except for monocytes and eosinophils, for which both relative and absolute counts showed a CV > 5%, (Table 1). For intra-assay, monocytes registered the highest CV for both relative (CV = 20.58%) and absolute counts (CV = 24.55%). For inter-assay, the monocytes registered the highest CV for relative counts (CV = 15.03%), and the eosinophils registered the highest CV for absolute counts (CV = 14.50%). In the study conducted on the HemoCue Hb 201, it was also possible to obtain a CV below 5% for intra- and inter-assay precision.

### 3.2. Linearity Results

**Figure 1:**
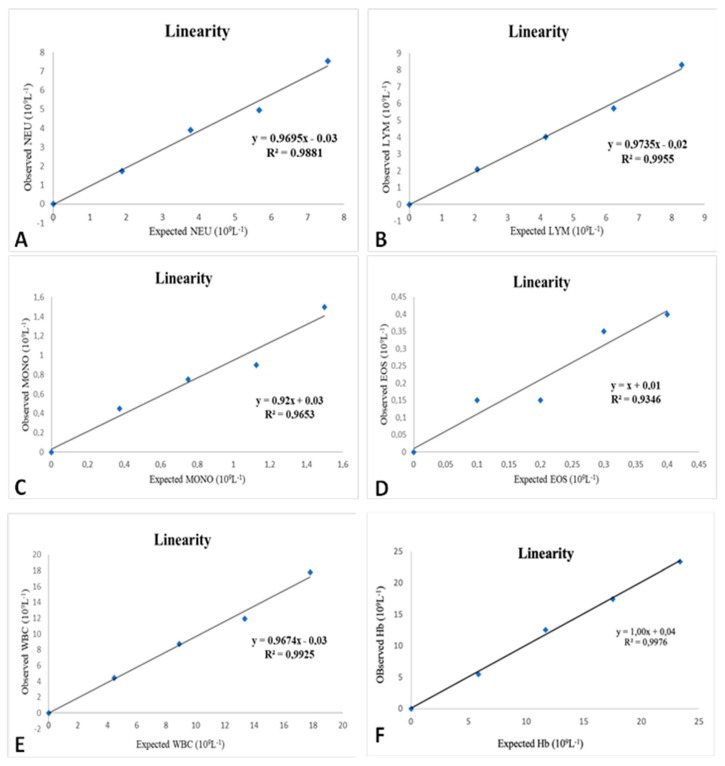
Linearity results for neutrophils (**A**), lymphocytes (**B**), monocytes (**C**), eosinophils (**D**), total leukocyte count (**E**), and hemoglobin (**F**). The observed values (*y*-axis) were compared with those expected (*x*-axis).

Linearity results are displayed in Table 2. The statistically significant results are represented through linear regressions in graphics A to F (Figure 1).

With the HemoCue WBC DIFF, the highest correlation coefficient was in the absolute neutrophil and lymphocyte count and the total leukocyte count r = 0.99 (*p* < 0.001). The parameter relative eosinophil count showed a correlation of r = 0.53. As for the HemoCue Hb 201, the correlation coefficient was r = 0.99.

For absolute neutrophil, lymphocyte, monocyte, and eosinophil counts and total leukocyte counts, the level of significance values was below 0.05, with the absolute lymphocyte count having the lowest value (*p* = 0.0001). For the relative count of neutrophils, lymphocytes, monocytes, and eosinophils, all the significance levels were above 0.05; the highest value was the relative count of eosinophils, with a *p* = 0.3618. In the measurement of hemoglobin in the HemoCue Hb 201, the significance value was below 0.05 (*p* = 0.0001).

The slope values for all parameters of both instruments were either one or very close to one, while the intercept values were zero or near zero. All linear regressions showed a high coefficient of determination (R2), with values exceeding 0.93. Linearity could not be calculated for basophils (Table 2).

### 3.3. Comparison Results

The results of the comparison between HemoCue WBC DIFF and HemoCue Hb 201 with ProCyte Dx are displayed in Table 3.

**Figure 2:**
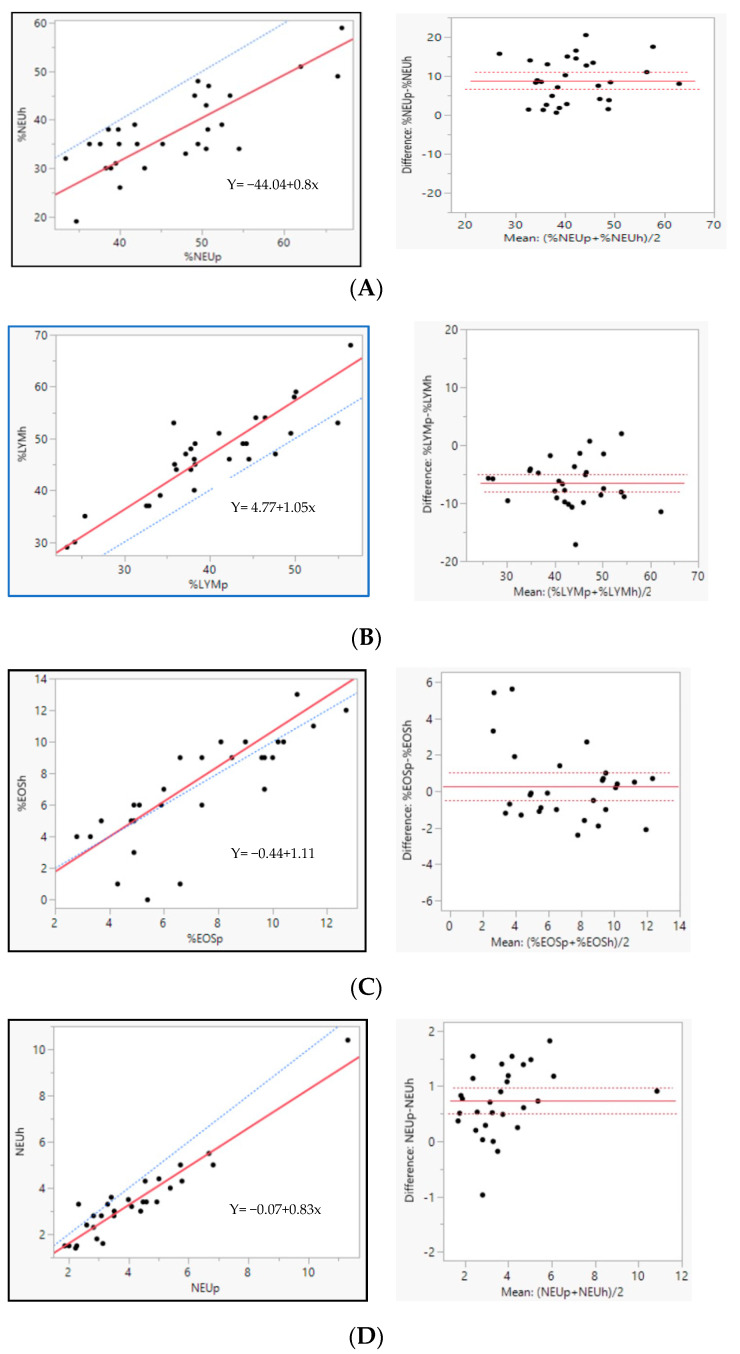

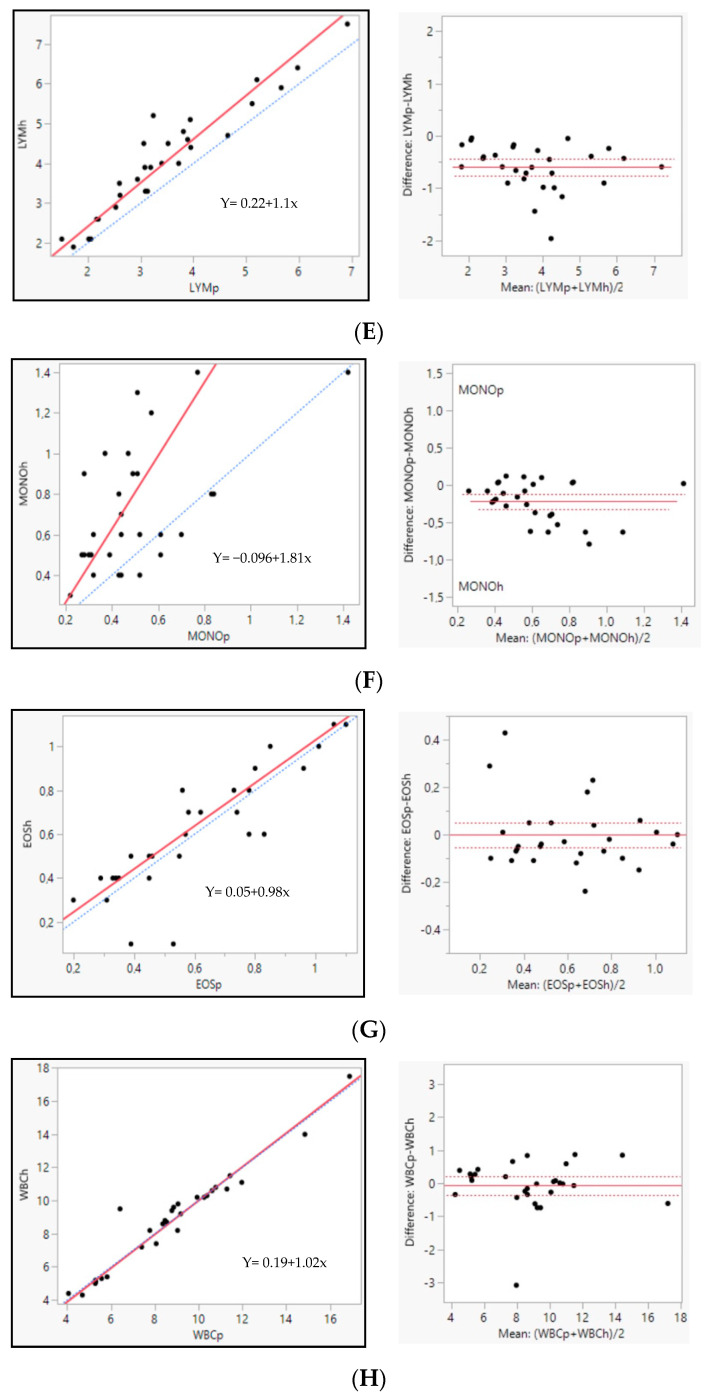

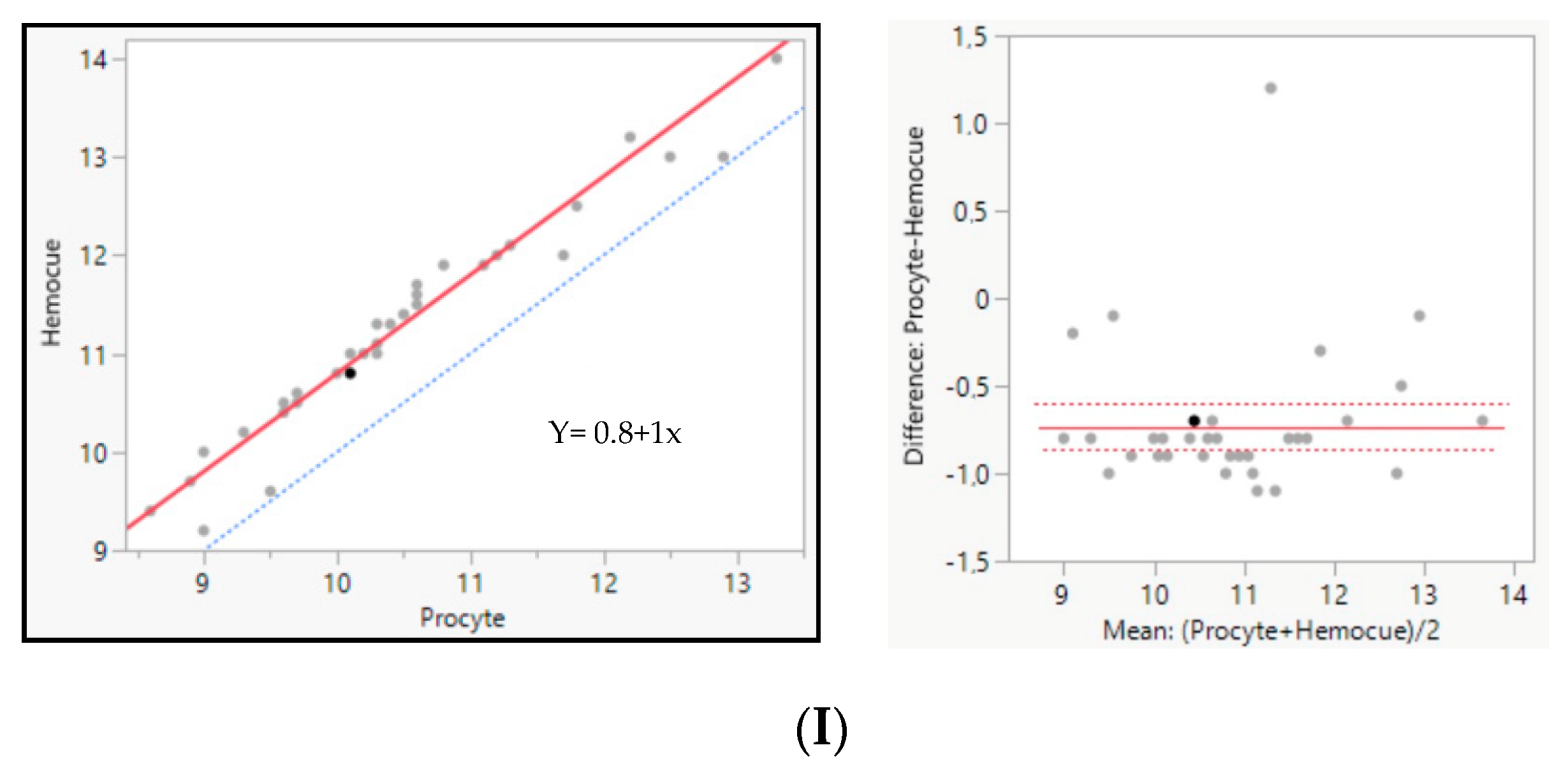
Agreement and correlation of the leucocyte differential and hemoglobin between HemoCue WBC DIFF, HemoCue Hb 201 and the ProCyte Dx. Left Passing–Bablok scatter diagrams—regression line in solid red, identity line (x = y) in dotted blue; Right—Bland-Altman diagrams, the difference between methods is plotted against the mean of both methods. The solid line represents the mean difference (bias) and the dotted line the ±1.96 standard deviation. Relative counts of neutrophils (NEU%) (**A**), lymphocytes (LYM%) (**B**), eosinophils (EOS%) (**C**); absolute counts of neutrophils (NEU) (**D**), lymphocytes (LYM) (**E**), monocytes (MONO) (**F**) eosinophils (EOS) (**G**), white blood cells absolute count (WBC) (**H**), and Hemoglobin (**I**).

For comparison of the HemoCue WBC DIFF with the ProCyte Dx, as seen in Table 3 and Figure 2, an excellent correlation coefficient was observed for WBC (r_s_ = 0.96), with a negative bias of 0.07%, as well as a lymphocyte absolute count ofr_s_ = 0.94 and a 0.6% bias. Neutrophils and eosinophils absolute counts presented a good correlation coefficient, r_s_ = 0.91 and r_s_ = 0.90, respectively, with a negative bias of 0.73% and 0, respectively. The lymphocyte and eosinophil relative count presented a good correlation coefficient, r_s_ = 0.84 and r_s_ = 0.86, with a bias of −6.55% and 0.27, respectively. The neutrophil relative count correlation was fair, r_s_ = 0.66, and a positive bias of 8.81% was observed. Monocyte and basophil relative and absolute counts demonstrated a poor correlation between both equipment’s results. The comparison between HemoCue Hb 201 and the ProCyte Dx was excellent, with a correlation coefficient of r_s_ = 0.94 and a negative bias of 0.73. Regarding the Passing–Bablok linear regression, it was not possible to trace a linear regression for basophil and monocyte relative counts and the basophil absolute count.

**Figure 3:**
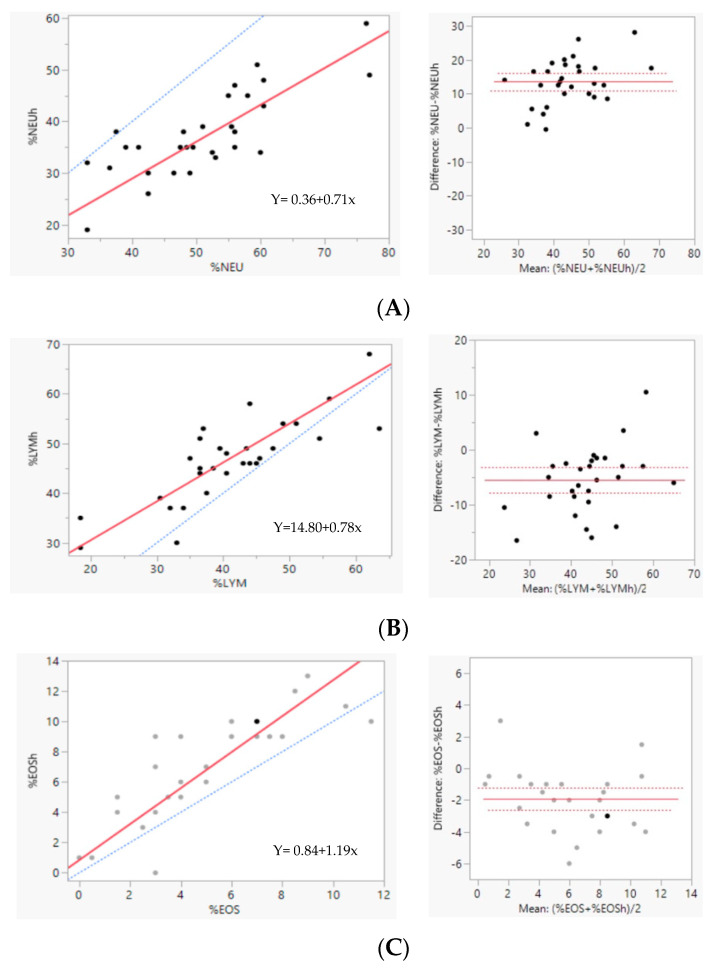
Agreement between manual method and HemoCue WBC DIFF results for leucocyte differential count. Left—Passing–Bablok scatter diagrams—regression line in solid red, identity line (x = y) in dotted blue; Right—Bland-Altman diagrams, the difference between methods is plotted against the mean of both methods. The solid line represents the mean difference (bias) and the dotted line the ±1.96 standard deviation. Neutrophils (NEU%) (**A**), eosinophils (EOS%) (**B**), and lymphocytes (LYM%) (**C**).

The results of the comparison between the manual differential count of leukocytes and the HemoCue WBC DIFF results are displayed in Table 4 and Figure 3. Lymphocytes and eosinophils absolute count presented a good correlation coefficient, r_s_ = 0.8 and r_s_ = 0.86, respectively, with a negative bias of 5.52% and 1.93%. The neutrophils relative count correlation was fair, r_s_ = 0.72, and a positive bias of 13.55% was observed. Monocytes and basophils demonstrated a poor correlation between manual and equipment results, r_s_ = −0.21 and r_s_ = 0.37, with bias of −5.69 and 0.23, respectively. Once again, regarding Passing–Bablok linear regression, it was not possible to trace a linear regression for monocytes and basophils.

## 4. Discussion

In this study, we validated the use of the HemoCue WBC DIFF and the HemoCue Hb 201b analyzers for hematological assessments in the Asinina de Miranda donkey, an endangered breed that often lacks access to veterinary facilities or laboratories. These analyzers are portable, low-maintenance, and suitable for use in field conditions due to their ease of handling and minimal training requirements [19,30]. Our results support their practicality for field-based veterinary clinics and emergency situations, as their performance demonstrated reliability in environments where more sophisticated equipment may not be available. The HemoCue WBC DIFF is a portable analyzer capable of measuring total and differential leukocyte counts, which is important for the early detection of inflammation or infection. According to the manufacturer, the average processing time for a sample is less than 5 min [9]. The HemoCue Hb 201 provides hemoglobin readings in under 1 min, which enables efficient screening for anemia, even in its early stages, where clinical symptoms may not yet be apparent. These features make both pieces of equipment suitable for field-based hematological assessments, as confirmed by their performance in this study. During the study, no maintenance was required for either device. However, we observed that proper handling of the microcuvette is critical, as underfilling or overfilling it can lead to inaccurate results, potentially resulting in misdiagnosis. These handling challenges have also been noted in previous studies [8,15].

Often, in veterinary medicine, hematology analyzers lack specific information for non-domestic animals such as wildlife, exotic animals, or even animals at risk of extinction. Consequently, veterinary hospitals, in the absence of a better option, analyze these animals using pre-existing reference values from other species, genera, or similar families [27,31]. This would not be problematic if the equipment could correctly distinguish each cell population, as morphology can differ between the species used as a reference and the species under study. Therefore, the obtained results might be imprecise, potentially leading to an erroneous clinical assessment of the animal, hence the importance of conducting an initial validation study to obtain precise and accurate results for a particular species in any circumstance [32].

In general, the HemoCue WBC DIFF and HemoCue Hb 201, when used with blood from animals of the Asinina de Miranda breed, presented very satisfactory results.

The HemoCue WBC DIFF demonstrated good precision in both intra- and inter-assay tests. This is a positive indicator of the equipment’s consistent reading ability to perform total counting and cell differentiation, showing no interference in its sample analysis capacity, even with the placement of a random laboratory sample between each run. The exceptions observed were in CV from absolute and relative counts of monocytes and eosinophils, in intra- and inter-assays, where the CV values were higher than the acceptable 5%. Since these populations of leukocytes present very low counts under physiological conditions, slight variations between manual and automated counts can lead to CV variations [25,33]. Bauer et al. [34] conducted a similar study for an intra-assay analysis using the Sysmex XT-2000 hematology analyzer on blood samples from horses. They observed similar results to those reported in the present study, where CVs were below 5% for relative and absolute counts of neutrophils and lymphocytes and the leukocyte total count. However, they presented CVs higher than 5% for relative (CV = 7.64% and 15.69%) and absolute counts (CV = 7.33% and 14.94%) of monocytes and eosinophils, respectively.

The HemoCue Hb 201 showed an intra-assay coefficient of variation (CV) of less than 3%, indicating good accuracy. In a previous study using HemoCue, CV values below 3% were also observed, further supporting the high accuracy demonstrated in this study [35]. The intra-assay accuracy of the earlier HemoCue-B model, used for measuring hemoglobin in horse blood, showed consistent results across successive measurements [36]. Similarly, our inter-assay results showed good CV values, aligning with findings from other studies that used the same equipment in different animal species [37,38], confirming the reliability of HemoCue in producing accurate results.

Concerning the linearity, the HemoCue WBC DIFF, in general, showed good results, with high and significant correlation values. However, the relative counts of neutrophils, lymphocytes, monocytes, and eosinophils exhibited non-significant values (*p* > 0.05), lacking statistical validity. The linearity of absolute and relative counts of basophils could not be calculated since the number of these cells was very low, even in the undiluted blood sample, making statistical analysis unattainable. This is because this population of leukocytes was present in very low numbers in the blood of healthy individuals, often resulting in mostly zero counts [18,27]. These results confirm the equipment’s capability to read, within its range, samples with both high and low concentrations, providing linear results for absolute counts of neutrophils, lymphocytes, monocytes, and eosinophils, as well as for the total leukocyte count. Regarding the relative leukocyte counts (%NEU, %LYM, %MONO, and %EOS), no explanation was found for the obtained results. Goldmann et al. [27] conducted a linearity study similar to the present one using horse blood samples. They obtained very good results for the absolute counts of neutrophils, lymphocytes, monocytes, and eosinophils, and for the total leukocyte count, with high r values, linear regression slopes very close to one, and linear regression intercepts close to 0, just like in the present study. This reinforces the reliability of the linearity of the HemoCue WBC DIFF analyzer for the mentioned parameters. The authors explained that basophil count statistics were not included in the study due to the low number of these cells, even in the most concentrated sample, which aligns with the findings in the present study.

Within the tested limits, the HemoCue Hb 201 was linear in parameter measurements in a graphically visible way, as seen in Figure 1. When we look at the statistics, the correlation coefficient obtained was excellent. In this study, an attempt was made to assess linearity by exceeding both the lower and upper limits designated by the manufacturer (0 g/dL and 25.6 g/dL, respectively) [13]. While the lower limit was reached during testing, the upper limit could not be tested. In practice, linearity is most important in samples with low hemoglobin values, as suspected cases of anemia typically involve low hemoglobin levels. Previous research showed that the earlier version, HemoCue B, is accurate for hemoglobin levels below 16 g/dL [36].

Manual techniques and the ProCyte Dx were used for comparison studies. Although the ProCyte Dx is not considered the gold standard, it is, to our knowledge, the only device validated for use with the Asinina de Miranda breed [22]. The comparison between the HemoCue WBC DIFF and the ProCyte Dx showed acceptable and significant results for most parameters, with the exception of absolute and relative counts of monocytes and basophils. A similar pattern was observed when comparing the HemoCue WBC DIFF with the manual method. Our findings align with a previous study on the ProCyte Dx, which demonstrated a good correlation for neutrophils and lymphocytes, an acceptable correlation for eosinophils, but a poor correlation for basophils and monocytes [22]. One explanation for the results obtained for monocytes and basophils, both in comparison with the ProCyte Dx and the manual method, is that these cells are present in low numbers in the blood of healthy animals [39]. As previously mentioned, small variations in their counts can have a greater effect on the statistical analysis compared to parameters with higher counts. Another possible explanation is the challenge of distinguishing large lymphocytes from monocytes. Like bovines and other animals, healthy Asinina de Miranda donkeys have a significant number of large lymphocytes, which may have led to the inaccurate characterization of some of these cells by either the studied equipment or the referenced methods [25].

Interestingly, a study on donkey blood comparing the IDEXX LaserCyte with the manual method found good agreement only for relative and absolute counts of monocytes and eosinophils, contrary to our findings [27]. Similarly, Silvestre-Ferreira et al. [39] conducted a comparative study using the IDEXX LaserCyte hematology analyzer and manual counting methods with blood samples from 29 horses. Their results showed a good correlation between both methods for the relative counts of neutrophils, lymphocytes, and eosinophils. However, like in our study, the absolute counts of monocytes and basophils showed a poor correlation. The authors suggested that these findings could be due to the low count of these cells in healthy horses, which supports our explanation. Additionally, variations in cell sizes and shapes in donkey blood, along with differences in analytical systems (such as dilution methods and dyes), may have influenced the results [27].

In our study, we were unable to find a clear explanation for the fair correlation in the comparison of neutrophil relative counts between the HemoCue WBC DIFF, the ProCyte Dx, and the manual method. This is particularly notable given that the comparison of absolute neutrophil counts between the HemoCue WBC DIFF and the ProCyte Dx was very good. Therefore, for clinical purposes, absolute counts should be prioritized over relative ones.

The comparison study results between the HemoCue Hb 201 and ProCyte DX showed excellent correlation. In another comparison study using a reference method (CELL DYN 2500) and two other hemoglobin measurement methods, HemoCue and i-STAT, the HemoCue demonstrated the highest correlation coefficient, indicating that its results were the most closely aligned with the reference method [35]. Harter et al. [21] and Posner et al. [40] reported reliable conclusions about the similarity of the values obtained with HemoCue compared to the reference method. Similarly, in our study, no statistically significant differences were observed between the HemoCue Hb 201 and the ProCyte DX, further reinforcing the consistency of using the HemoCue Hb 201 for hemoglobin determination in the Asinina de Miranda breed.

This study has some limitations, the most significant being the limited number of samples used for comparison studies. Additionally, the total manual WBC count or hemoglobin concentration using the cyanmethemoglobin method could not be determined because the samples were collected as part of a seasonal prophylactic campaign. The large volume of samples collected each day made it impractical to perform manual techniques, and the distance from the animals’ location to the main laboratory, several hours away, made it impossible to complete the analysis within an acceptable time frame.

The ASVCP established guidelines for quality assurance for the use of POCT systems in veterinary medicine, and interested readers are referred to this resource for more details. Briefly, there should be proper blood sample handling; blood smear reviews should be performed anytime there is suspicion that automated WBC differential counts are inaccurate; periodic quarterly comparability testing is recommended to confirm instrument function, reagent stability, and comparability to an appropriate peer group or reference laboratory [7].

## 5. Conclusions

The HemoCue WBC DIFF and HemoCue Hb 201 demonstrated excellent performance without requiring maintenance during the study, making them reliable, low-maintenance devices for field veterinary use. Their portability, speed, and ability to operate without a power source further enhance their suitability for veterinary diagnostics. When compared to the bench-top ProCyte Dx and a manual reference method, no significant differences were found in the clinical diagnosis of Asinina de Miranda donkeys. Overall, both systems provided reliable results for total and differential WBC counts and hemoglobin measurements in this breed.

## Figures and Tables

**Table 1:** Coefficients of variation calculated intra- and inter-assay precision.

Parameters		Intra-Assay (*n* = 20)	Inter-Assay (*n* = 5)
HemoCue WBC DIFF		CV%	Average CV%
	NEU	4.44	4.44
	LYM	4.28	4.94
	MONO	24.55	14.28
	EOS	15.31	14.50
	BASO	0.00	0.00
	WBC	2.85	3.06
	%NEU	4.76	4.65
	%LYM	4.64	4.73
	%MONO	20.58	15.03
	%EOS	15.68	12.56
	%BASO	0.00	0.00
HemoCue Hb 201	Hb	1.98	4.07

NEU: Absolute counts of neutrophils (10^9^ L^−1^); LYM: Absolute counts of lymphocytes (10^9^ L^−1^); MONO: Absolute counts of monocytes (10^9^ L^−1^); EOS: Absolute counts of eosinophils (10^9^ L^−1^); BASO: Absolute counts of basophils (10^9^ L^−1^); %NEU: Relative counts of neutrophils; %LYM: Relative counts of lymphocytes; %MONO Relative counts of monocytes; %EOS: Relative counts of eosinophils; %BASO: Relative counts of basophils; Hb—Measurement of the amount of hemoglobin (g/dL); n: Number of determinations; CV—Coefficient of variation.

**Table 2:** Results for linearity using 5 levels of dilution.

Parameters (*n* = 5)		Correlation Coefficient (r)	Significance Level (*p*)
HemoCue WBC DIFF	NEU	0.99	0.0006
	LYM	0.99	0.0001
	MONO	0.98	0.0028
	EOS	0.97	0.0072
	WBC	0.99	0.0003
	%NEU	0.73	0.1576
	%LYM	0.71	0.1753
	%MONO	0.58	0.3063
	%EOS	0.53	0.3618
HemoCue Hb 201	Hb	0.99	0.0001

NEU: Absolute counts of neutrophils (10^9^ L^−1^); LYM: Absolute counts of lymphocytes (10^9^ L^−1^); MONO: Absolute counts of monocytes (10^9^ L^−1^); EOS: Absolute counts of eosinophils (10^9^ L^−1^); %NEU: Relative counts of neutrophils; %LYM: Relative counts of lymphocytes; %MONO: Relative counts of monocytes; %EOS: Relative counts of eosinophils; Hb: Measurement of the amount hemoglobin (g/dL); N: Number of determinations; r: correlation coefficient.

**Table 3:** Agreement and correlation of the leucocyte differential count and hemoglobin between HemoCue WBC DIFF, HemoCue Hb 201 and the ProCyte Dx.

Equipment	Parameters	Spearman’s Rho (r_s_)	Bias (95% Limits of Agreement)
HemoCue WBC DIFF *n* = 29	**NEU %**	0.66	8.81 (6.65 to 10.97)
	**LYM %**	0.84	−6.55 (−8.08 to −5.02)
	**MONO %**	−0.17	−2.40 (−3.34 to −1.47)
	**EOS %**	0.86	0.27 (0.48 to 1.02)
	**BASO %**	−0.17	0.53 (0.31 to 0.76)
	**WBC**	0.96	−0.07 (−0.35 to 0.22)
	**NEU**	0.91	0.73 (0.50 to 0.97)
	**LYM**	0.94	0.6 (−0.76 to −0.43)
	**MON**	0.5	−0.22 (−0.32 to −0.12)
	**EOS**	0.90	−0.00 (−0.05 to 0.05
	**BASO**	−0.16	0.04 (0.02 to 0.051)
HemoCue Hb 201 *n* = 39	**Hb**	0.94	−0.73 (−0.86 to −0.60)

NEU: Absolute counts of neutrophils (10^9^ L^−1^); LYM: Absolute counts of lymphocytes (10^9^ L^−1^); MONO: Absolute counts of monocytes (10^9^ L^−1^); EOS: Absolute counts of eosinophils (10^9^ L^−1^); BASO: Absolute counts of basophils (10^9^ L^−1^); NEU %: Relative counts of neutrophils; LYM %: Relative counts of lymphocytes; MONO %: Relative counts of monocytes; EOS %: Relative counts of eosinophils; BASO %: Relative counts of basophils; Hb: Measurement of the amount of hemoglobin (g/dL); n: Number of cases.

**Table 4:** Agreement and correlation of the leucocyte differential count between HemoCue WBC DIFF and manual method.

Parameters (*n* = 29)	Spearman’s Rho (r_s_)	Bias (95% Limits of Agreement)
NEU %	0.72	13.55 (11.02 to 16.08)
LYM %	0.8	−5.52 (−7.8 to −3.24)
MONO %	−0.21	−5.69 (−6.75 to −4.63)
EOS %	0.86	−1.93 (−2.64 to −1.23)
BASO %	0.37	0.23 (0.09 to 0.38)

NEU %—Relative counts of neutrophils; LYM %—Relative counts of lymphocytes; MONO %—Relative counts of monocytes; EOS %—Relative counts of eosinophils; BASO %—Relative counts of basophils.

## Data Availability

All the data supporting the results are included in the manuscript. The data set is available from the corresponding author on reasonable request.
